# The case of an 85‐year‐old woman with subacute onset of bilateral chorea

**DOI:** 10.1002/acn3.52060

**Published:** 2024-05-03

**Authors:** Nathan J. Nakatsuka, Vihang Nakhate, Daniel S. Harrison, Kristin M. Galetta, Abby L. Olsen

**Affiliations:** ^1^ Harvard Medical School Boston Massachusetts USA; ^2^ Department of Neurology Brigham and Women's Hospital Boston Massachusetts USA; ^3^ Department of Neurology Massachusetts General Hospital Boston Massachusetts USA; ^4^ Department of Neurology University of Pittsburgh Pittsburgh Pennsylvania USA; ^5^ Department of Neurology UPMC Pittsburgh Pennsylvania USA

## Summary of Case (HPI, Relevant Examination Findings, and Relevant Data)

An elderly woman presented with subacute onset of chorea following a hospitalization for severe, uncontrolled diabetes, a urinary tract infection, and 6 months of depression. Her neurological examination demonstrated bilateral chorea involving the arms, legs, and jaw. MRI of the brain demonstrated bilateral T1 hyperintensity. She was diagnosed with diabetic striatopathy, also known as nonketotic hyperglycemic chorea, a rare complication of diabetes mellitus that classically causes hemichorea in the setting of very high blood glucose without ketosis. This case demonstrates typical imaging findings of diabetic striatopathy despite several atypical clinical features, including bilateral chorea, development of symptoms weeks after improvement in blood glucose, and demographics of the patient.

## Diagnosis

Diabetic striatopathy.^1^, ^2^

## Take‐Home Points

**Figure 1:**
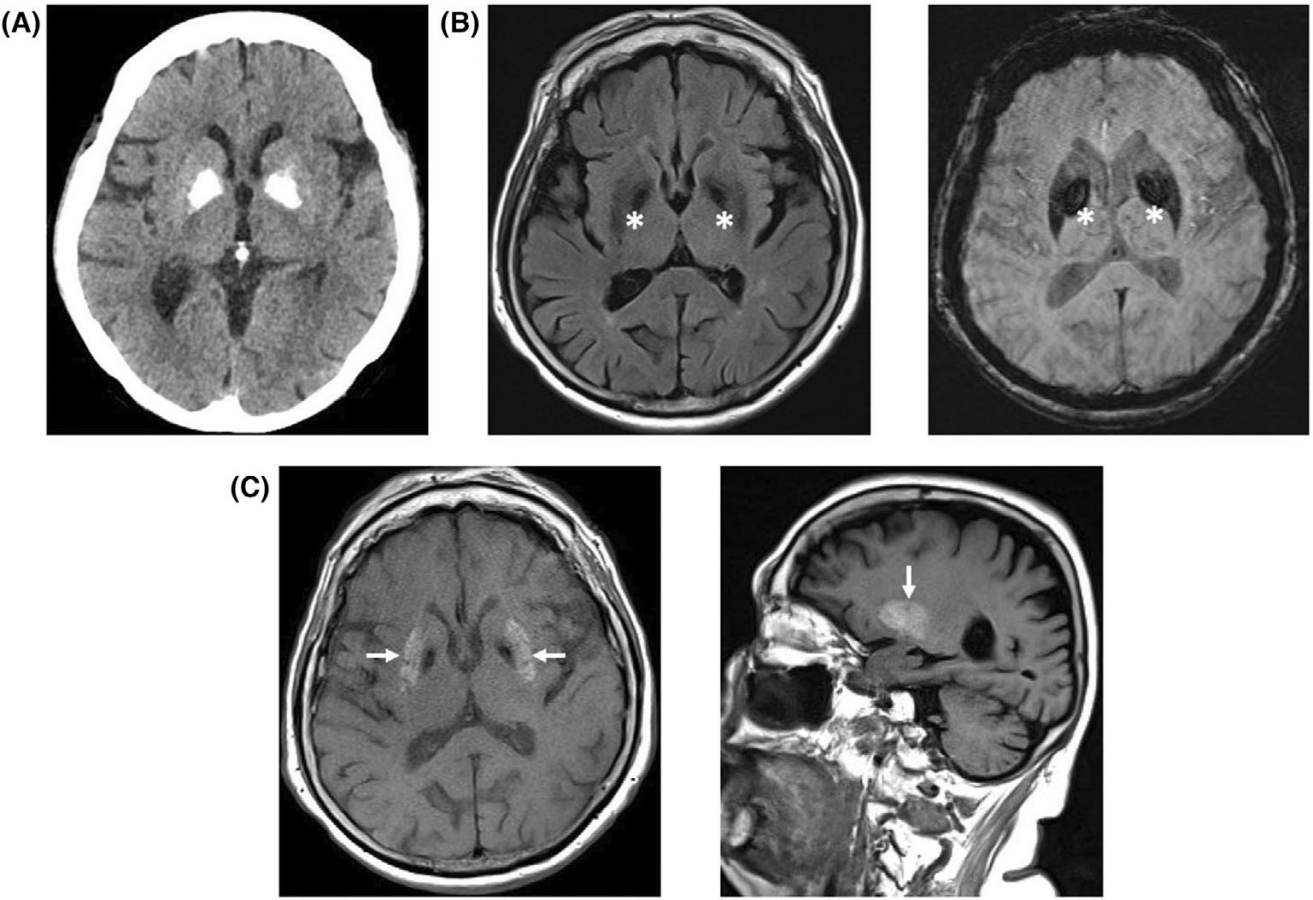
CT head and MRI brain showing globus pallidus calcifications and putaminal T1 hyperintensity. (A) Non‐contrast head CT axial image showing bilateral hyperdensities in the globus pallidi consistent with age‐related calcification, unchanged from CT head 1 year prior (not shown). (B) MRI brain axial T2 FLAIR sequence (left) showing T2 hypointensities in bilateral globus pallidi (indicated by white asterisk) corresponding to hyperdensities on CT; SWI sequence (right) showing susceptibility artifact in bilateral globus pallidi (indicated by white asterisk) corresponding to CT hyperdensities, consistent with age‐related calcification. (C) MRI brain axial (left) and sagittal (right) T1 pre‐contrast sequences showing hyperintensity of bilateral putamen (indicated by white arrows).

- Diabetic striatopathy, or nonketotic hyperglycemia induced chorea, occurs in the setting of markedly elevated blood glucose.
- The mainstay of treatment is lowering of blood glucose, though, as our case indicates, chorea can start after this occurs.
- If normalization of glucose does not resolve symptoms, additional treatments include neuroleptics and benzodiazepines.
- The typical imaging finding of diabetic striatopathy is T1 hyperintensity in the putamen (Fig. 1).

## References

[1] Lee SH , Shin JA , Kim JH , et al. Chorea‐ballism associated with nonketotic hyperglycaemia or diabetic ketoacidosis: characteristics of 25 patients in Korea. Diabetes Res Clin Pract. 2011;93:e80‐e83. 21632136 10.1016/j.diabres.2011.05.003

[2] Chua CB , Sun CK , Hsu CW , Tai YC , Liang CY , Tsai IT . “Diabetic striatopathy”: clinical presentations, controversy, pathogenesis, treatments, and outcomes. Sci Rep. 2020;10(1):1594. 32005905 10.1038/s41598-020-58555-w PMC6994507

